# Automatic 3D Postoperative Evaluation of Complex Orthopaedic Interventions

**DOI:** 10.3390/jimaging9090180

**Published:** 2023-08-31

**Authors:** Joëlle Ackermann, Armando Hoch, Jess Gerrit Snedeker, Patrick Oliver Zingg, Hooman Esfandiari, Philipp Fürnstahl

**Affiliations:** 1 Research in Orthopedic Computer Science, Balgrist University Hospital, University of Zurich, 8008 Zurich, Switzerland; 2 Laboratory for Orthopaedic Biomechanics, ETH Zurich, 8093 Zurich, Switzerland; 3 Department of Orthopedics, Balgrist University Hospital, University of Zurich, 8008 Zurich, Switzerland

**Keywords:** orthopaedic computer science, cut detection, postoperative evaluation, machine learning, deep learning, segmentation

## Abstract

In clinical practice, image-based postoperative evaluation is still performed without state-of-the-art computer methods, as these are not sufficiently automated. In this study we propose a fully automatic 3D postoperative outcome quantification method for the relevant steps of orthopaedic interventions on the example of Periacetabular Osteotomy of Ganz (PAO). A typical orthopaedic intervention involves cutting bone, anatomy manipulation and repositioning as well as implant placement. Our method includes a segmentation based deep learning approach for detection and quantification of the cuts. Furthermore, anatomy repositioning was quantified through a multi-step registration method, which entailed a coarse alignment of the pre- and postoperative CT images followed by a fine fragment alignment of the repositioned anatomy. Implant (i.e., screw) position was identified by 3D Hough transform for line detection combined with fast voxel traversal based on ray tracing. The feasibility of our approach was investigated on 27 interventions and compared against manually performed 3D outcome evaluations. The results show that our method can accurately assess the quality and accuracy of the surgery. Our evaluation of the fragment repositioning showed a cumulative error for the coarse and fine alignment of 2.1 mm. Our evaluation of screw placement accuracy resulted in a distance error of 1.32 mm for screw head location and an angular deviation of 1.1° for screw axis. As a next step we will explore generalisation capabilities by applying the method to different interventions.

## 1. Introduction

The rapid technological advancements in recent years and their increased adoption in medical fields such as orthopaedic surgery, has led to numerous innovations in areas such diagnosis [1,2], surgical robotics [3] and intraoperative navigation [4,5,6,7,8,9,10,11,12,13,14]. Since three-dimensional (3D) preoperative planning is a fundamental requirement for surgical navigation systems and surgical robotics, significant amount of research has been dedicated to automating the planning process [15,16,17,18,19,20,21,22]. Analysing whether the preoperative plan was implemented successfully during the intervention is of equal importance. However, due to the lack of automated methods, 3D postoperative outcome evaluation is not yet used in clinical practise to date. State-of-the-art postoperative evaluation is based on 2D-imaging and patient related outcome measures. Although 3D outcome evaluation is a particularly powerful tool, it is technically demanding and can take up to 6 h due to the lack of adequate automatic methods [23].

**Figure 1:**
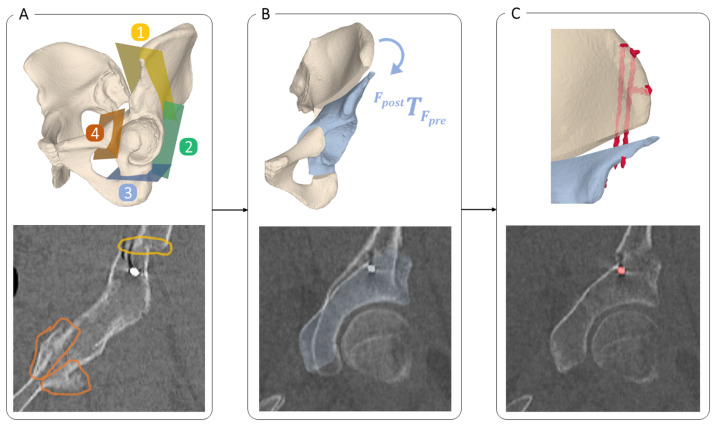
Overview of the three common surgical steps in orthopaedic interventions on the example of the Periacetabular Osteotomy (PAO): (**A**) Cutting Bone: Four osteotomies are performed, namely the ischial (1), pubic (2), supra- (3) and retroacetabular (4) osteotomy, to mobilise the acetabular fragment (in blue). (**B**) Anatomy Manipulation and Repositioning: Repositioning of the acetabular fragment (in blue) to restore the physiological anatomy. The transformation ${}^{F_{post}}T_{F_{pre}}$ represents the relative repositioning of the fragment from $F_{pre}$ to $F_{post}$, where $F_{pre}$ and $F_{post}$ are the respective local coordinate systems of the cropped pre- and postoperative CT images. (**C**) Implant Placement: Fixing the acetabular fragment in its new position using screw implants (red). The bottom row shows slices of a postoperative CT: In (**A**) the supraacetabular (in yellow) and pubic (in orange) osteotomies are highlighted. An overlap of pre- and postoperative CTs is shown in (**B**), to indicate the acetabular transformation. Finally, in (**C**), the cross section of a screw is visible in red.

We propose a fully automatic method for 3D postoperative quantification of the most common steps of an orthopaedic intervention: A: Cutting Bone (i.e., performing an osteotomy), B: Anatomy Manipulation and Repositioning and C: Implant Placement (e.g., screws, plates, prosthesis). To investigate the feasibility of the proposed approach, one of the most complex orthopaedic interventions called Periacetabular Osteotomy of Ganz (PAO) [24] was used as the target intervention in this study, which includes all of the aforementioned surgical steps (Figure 1).

**Figure 2:**
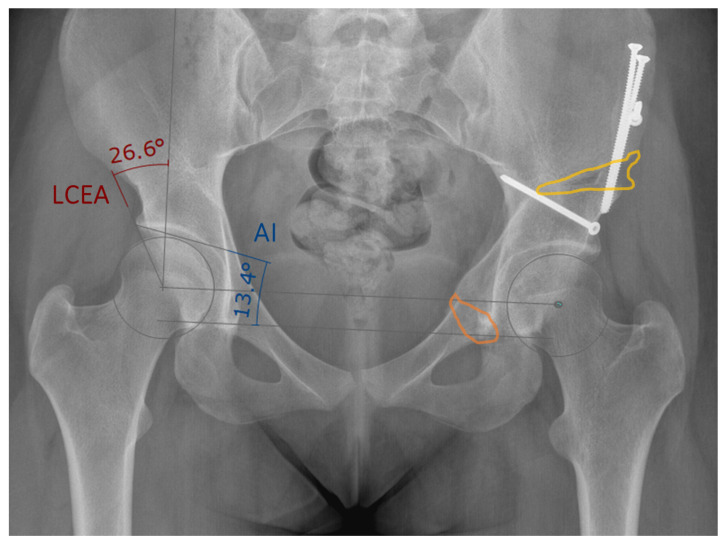
Example of a postoperative AP pelvic radiograph after PAO, showing the lateral center-edge angle (LCEA) and acetabular index (AI) measurement. On the left hip joint, the area where the supraacetabular oteotomy has been performed is shown in yellow. The area of the pubic osteotomy is marked in orange.

PAO is a hip surgery, typically performed in young patients who suffer from a condition called residual hip dysplasia. Residual hip dysplasia is characterised by insufficient acetabular coverage of the femoral head, causing hip pain and possibly early onset of osteoarthritis. The PAO involves four pelvic osteotomies namely the supra- (1), retroacetabular (2), ischial (3) and pubic (4) osteotomy, which separate the acetabular fragment from the remaining pelvic bone (see Figure 1(A1–A4), respectively). The mobile fragment (Figure 1, in blue) is then rotated to its new position and fixated using 3 to 4 screws. Postoperative evaluation after PAO therefore entails quantifying all 4 osteotomy planes represented by a 3D point and 3D normal vector (Figure 1A) in a 4 × 4 transformation matrix encoding 3D orientation and position of the fragment (Figure 1B) and lastly, determining the screw positions represented as a 3D point and a 3D normal vector (Figure 1C). Although PAO is a complex intervention, conventional postoperative outcome evaluation are limited to mainly two radiographic (2D) parameters, being the center-edge (LCEA) angle of Wiberg and the acetabular index (AI) angle of Tonnis [25]. A LCEA angle of 23°–33° and AI angle of 2°–14° are typically considered healthy [26,27]. Figure 2 shows a postoperative X-ray after PAO (left hip), with an LCEA angle of 26.6° and AI angle of 13.4° on the healthy side.

Besides Hoch et al. [23], different 3D approaches for postoperative outcome evaluation have been presented but none were sufficiently comprehensive and automated to a level where they could be used in clinical practice. Manual 3D outcome evaluation was extensively used in studies on computer-assisted deformity correction to assess bone, implant and osteotomy parameters in the wrist [28,29], forearm [30,31,32], shoulder [33,34], knee [35,36,37,38,39] and foot [40,41,42]. Beside these manual approaches, automatic methods have also been developed. Murphy et al. [4] pusblished a clinical evaluation of a biomechanical guidance system for PAO in 2016, where they report preoperative planning, intraoperative navigation as well as the postoperative evaluation of the 3D acetabular realignment. To evaluate their system, the authors aligned pre- and postoperative CT scans through image registration using normalised mutual information (NMI) as metric. Kyo et al. [43] evaluated implant orientation in postoperative CT images after total hip arthroplasty, by overlaying a 3D model of the implant on a postoperative CT image of the implant. In 2022, Gubian et al. [44] evaluated CT-navigated pedicle screw placement, by comparing the preoperative trajectory plan with the corresponding postoperative screw position, determined by manual segmentation of the postoperative CT. Uozumi et al. [45] proposed an automatic 3D evaluation of screw placement after anterior cruciate ligament reconstruction using multidetector CT images. Their method consisted of thresholding the images to isolate the screw voxels, based on which the center of mass of the screws were found. The corresponding screw center lines were detected through Principal Component Analysis (PCA) [46]. In 2018, Esfandiari et al. [47] reported a deep learning based technique for screw position assessment on intraoperative X-rays of the spine anatomy. First, a convolutional neural network was utilised to classify every pixel into three distinct classes, namely the screw head, screw shaft, and background. Subsequently, a skeletonisation algorithm was applied to extract the central axis. To date, no studies have been reported in literature regarding the quantification of osteotomies, but the field of automatic fracture detection attempts to solve a similar problem. Several studies have investigated fracture detection using deep learning [48,49,50,51,52,53,54,55,56,57,58,59,60,61,62,63,64,65]. Studies on fracture detection have mainly been focusing on the classification task (fracture/no fracture), such as Tomita et al. [62] In 2018, Tomita et al. [62] published a pre-screening system to improve osteoporotic vertebral fractures (OVFs) diagnosis on chest, abdomen and pelvis CTs, flagging suspicious cases prior to review by radiologists. Cuts were localised by predicting bounding boxes around the area of fracture. Lindsey et al. [59], developed a deep learning approach for fracture detection and localisation on wrist radiographs. The model had two outputs, a binary classification for fracture detection and a probability map (heat map) showing the confidence at each pixel location for it to be part of a given fracture. They found that the detection of wrist fractures by clinicians improved significantly, when provided with the assistance of the trained model [59]. The localisation of fractures was not quantified, but served as an assistance for clinicians to find a potential fracture. In 2022, Joshi et al. [48] presented the first fracture detection and localisation method on wrist radiographs, which in addition to detection and localisation, also provided a segmentation mask of the fractures using instance segmentation with a modified version of mask R-CNN [66] architecture.

As per our knowledge, the approach presented in this study is the first work introducing a fully automatic method for 3D postoperative outcome evaluation. Our technical contributions are:

- The first fully automatic 3D measurement method of bone cut accuracy is presented.
- Thanks to our cut detection method, our combined segmentation and registration approach measures anatomy manipulation and repositioning automatically and accurately, even in the presence of bone in-growth and callus.
- Lastly, an accurate and fully automatic 3D screw placement quantification method is presented.

Our approach was evaluated on 27 PAO interventions and compared against a manually performed 3D outcome evaluation method [23].

## 2. Materials and Methods

In the following, we first provide details on our data collection protocol in Section 2.1 and later describe the proposed automatic outcome evaluation method in Section 2.3. Section 2.3 is organised in three subsections: Osteotomy detection and quantification is discussed in Section 2.3.1, quantification of anatomy repositioning in Section 2.3.2 and implant quantification in Section 2.3.3. The following mathematical notations are used throughout this article: The global coordinate system of the pre- and postoperative CT images are denoted as $CT_{pre}$ and $CT_{post}$ respectively. The local coordinate system of the cropped pre- and postoperative CT images are denoted as $F_{pre}$ and $F_{post}$ respectively. A transformation from $CT_{pre}$ to $CT_{post}$ is denoted as ${}^{CT_{post}}T_{CT_{pre}}$. The relative repositioning of the fragment is described by the transformation from $F_{pre}$ to $F_{post}$ and denoted as ${}^{F_{post}}T_{F_{pre}}$.

### 2.1. Patient Selection and Imaging

This study included 27 patients (9 m, 18 f) who underwent PAO in our institution between March 2018 and May 2020. This study was approved by the responsible ethical committee (approval number: BASEC-Nr. 2018-01921). The mean age of the subjects was 25 years (14–33 y). 14 patients underwent surgery of the right hip joint, 13 of the left. Three patients had already undergone PAO on the contralateral side previous to this study. For two patients both hips were included, since their PAO interventions on both sides were performed during the mentioned time frame. Exclusion criteria were a previous hip surgery other than PAO (i.e., total hip replacement), a data set which is incomplete or does not comply to the CT imaging protocol. For all patients, pre- and postoperative computed tomography (CT) scans of the pelvis were acquired according to a standard protocol of the radiology department of Balgrist University Hospital. The radiographic assessment was performed pre- and 15.2 ± 3.4 weeks postoperatively, using a 64-detector row Somatom Edge CT^®^ device (Siemens, Erlangen, Germany). The slice thickness was 1.0 mm and the in-plane resolution (x-y) was 0.4 × 0.4 mm. The images were resampled to shape [128,128,128] for all steps that involved Deep Learning. After resampling, the full pelvis CT images had a voxel size of 2.86 mm and the cropped images had a voxel size of 1 mm. 3D models of the pelves were extracted using the global thresholding and region growing functionalities of a commercial segmentation software (Mimics medical, Materialise NV, Leuven, Belgium) [8,34].

### 2.2. Manual 3D Postoperative Evaluation

Manual 3D postoperative evaluation is based on a pipeline that involves numerous manual steps. On the example of PAO, the overall process can be summarised as follows. The segmentation of the bone models was performed for both pre- and postoperative CT images. Segmentation of the post-operative CT images still relies on extensive manual refinement as the presence of metal artifacts make automatic segmentation methods less effective [67]. The 3D assessment of the osteotomies is the most challenging and inaccurate part due to the formation of callus resulting from the bone healing process. Callus is a visible irregularity on postoperative bone, similar to scarring in soft tissue. For each of the four osteotomies, a plane object was placed on the postoperative bone model at the location of the callus. Its position on the bone model was verified by looking at the model from multiple perspectives. Once the position of each osteotomy plane was determined, the cuts were simulated on the 3D postoperative bone model to free the mobile fragment in-silico. To identify the performed intraoperative anatomy repositioning (Figure 1B), the mobile fragment was aligned with the preoperative bone model using surface registration (ICP) [68] followed by manual fine-tuning. Finally, to determine the positions of the screws, manual selection of two points was carried out on the segmented screw models, one at the head and the other at the tip of each screw.

### 2.3. Computer-Assisted 3D Postoperative Evaluation

**Figure 3:**
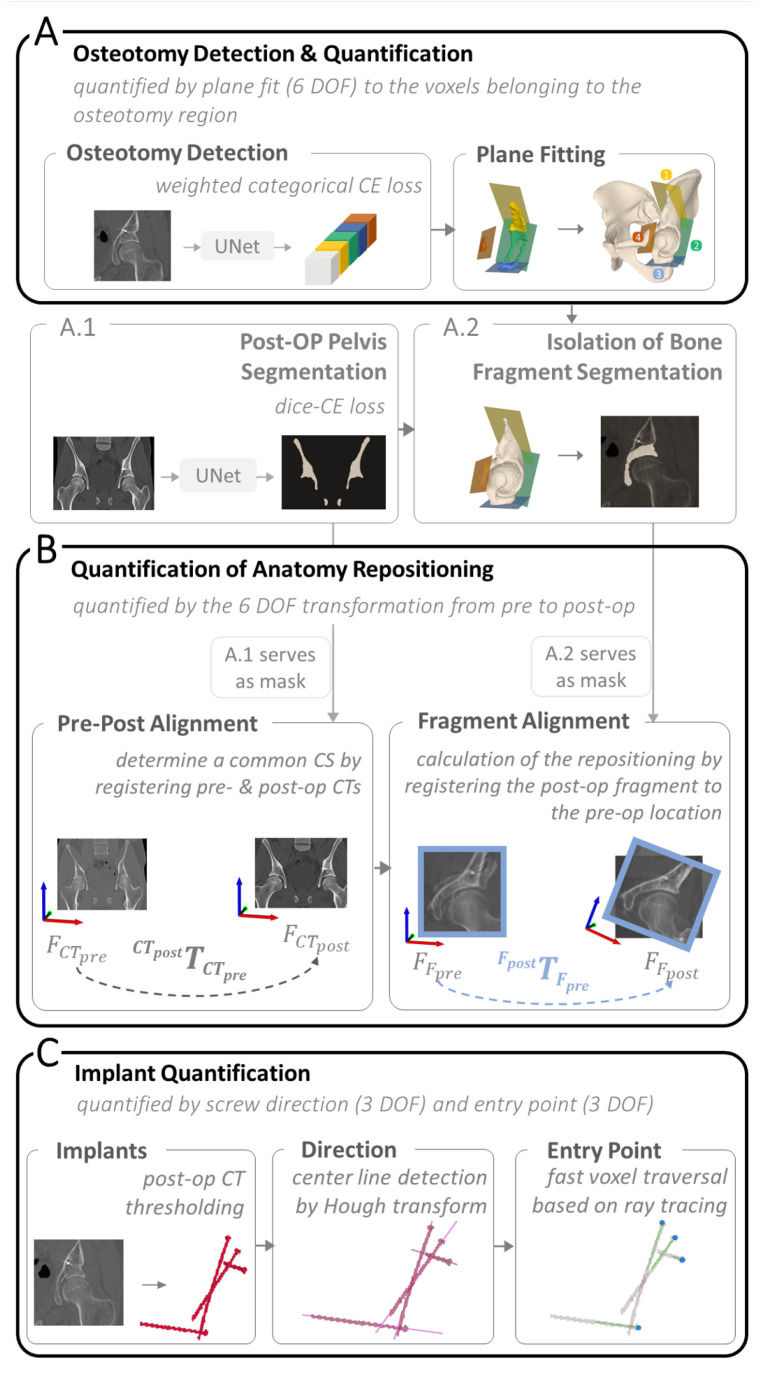
An overview of the proposed pipeline consisting of three main components: (**A**) Osteotomy Detection and Quantification, where the four osteotomy planes (1–4) are shown in yellow, green, blue and red. (**A.1**,**A.2**) represent how the two registration masks, used in (**B**), were created. (**B**) Quantification of Anatomy Repositioning and (**C**) Implant Quantification.

Figure 3 provides a high-level overview of our method, where A describes osteotomy detection and quantification, B shows the quantification of anatomy repositioning and C illustrates the three steps towards implant quantification. In A, we trained a network to specifically identify all voxels belonging to the cut region of each osteotomy (Section 2.3.1). A 3D plane was then fitted to each segmented cut region to quantify the osteotomy. In B, anatomy repositioning is quantified by two consecutive registrations, first a coarse alignment between pre- and postoperative CT, followed by a registration of the fragment from the post- to the preoperative position. We used two registration masks to specify the region of interest. The first registration mask for the pre-post alignment was inferred by a bone segmentation network to segment the full pelvis (A.1). Based on the plane positions predicted in A, the second registration mask for the fragment alignment was created (A.2), by isolating the area of the acetabular fragment in the full pelvis segmentation (A.1). The screw locations were determined in three steps, illustrated in C. First, the post-op CT was thresholded to obtain point clouds of the screws. Second, the screw center lines were determined by applying Hough Transform [69]. The last step was finding the screw head locations achieved by fast voxel traversal for ray tracing [70]. In the following chapters, the details on implementation are reported.

#### 2.3.1. Osteotomy Detection and Quantification

The basis for calculating the planes was a pixel-wise identification of the osteotomy areas. For this purpose, a 3D multi-label segmentation network was used. The input of the network were the postoperative CT images, which were previously pre-processed by normalisation and cropping them around the hip joint, such that the full fragment was included and then resampled to [128,128,128]. The output tensor was of shape [128,128,128,5] consisting of the background [128,128,128,0] as well as one dimension per osteotomy [128,128,128,i],i∈[1,4], where channel i corresponds to the ith osteotomy plane of Figure 1A. The network was trained during 40 epochs and had a 3D UNet [71] structure consisting of five blocks of convolutional layers with 3 × 3 × 3 filters, followed by a max-pooling layer, where the number of filters were doubled for each convolutional block. We used 16 filters for the first convolutional block and doubled the number of filters for each block. Adam’s algorithm was used as the optimizer [72]. The activation function for all convolutional layers was leaky ReLU, except for the last layer which was softmax. The training data was manually annotated using pixel-wise segmentation and included the bony areas identified as part of the cut, using the callus formation. For epoch 0 to 10, the learning rate $lr=1\cdot e^{-4}$, after that, it was changed to $1\cdot e^{-5}$. The 27 postoperative images were augmented offline resulting in 405 input images. Augmentation consisted of at least one or a combination of vertical flipping, translation or rotation. To train this network, a weighted categorical cross-entropy loss $L_{WCCE}$ was used:

(1) $$L_{WCCE}=-\sum_{i=1}^{i=N}t_{i}\cdot log\left(p_{i}\right)\cdot w_{i}$$

where *N* denotes the number of classes. The weights were empirically determined to be w=[10,270,260,270,260].

The segmentation obtained from the network served as the basis for plane fitting, which was performed for each identified cut region using principal component analysis (PCA) [46], taking the smallest eigenvector as the plane normal and the center-of-mass as the plane center.

#### 2.3.2. Quantification of Anatomy Repositioning

To quantify anatomy repositioning, we propose a masked multi-step registration approach which entailed a coarse alignment of the pre- and postoperative CT images followed by a fine fragment alignment of the repositioned anatomy.

Coarse alignment: The first registration calculates the transformation ${}^{CT_{post}}T_{CT_{pre}}$ used to superimpose the pre- with the postoperative CT images (Figure 3B, Pre-Post Alignment). To this end, the pelvis bone was utilised as a common reference between pre- and postoperative CT images and used as registration mask for the coarse alignment (Figure 3(A.1)). The mask $M_{coarse}$ was obtained by applying deep learning segmentation using the same network architecture as for the osteotomy detection described in Section 2.3.1, whereas learning rates, input/output images, activation function as well as loss function, differed. For epoch 1 to 20, the learning rate was set to $1\cdot e^{-4}$, for epoch 20 to 30 it was $1\cdot e^{-5}$ and between 30 and 40 it was $1\cdot e^{-6}$. Input and output size were both [128,128,128] and sigmoid was used as activation function for the final layer. The network was trained for 40 epochs. To make the network robust against the presence of implants and metal artefacts, the dataset consisted of not only 25 preoeprative CTs but also 27 postoperative CTs with implants. We augmented them offline to a total of 520 images and randomly applying either one or a combination of vertical flipping, rotation or translation to the images. Dice-CE Loss [73] $L_{DCE}$ was used as the loss function and was defined as:

(2) $$L_{DCE}=\left(1-\alpha \right)\cdot L_{CE}+\alpha \cdot L_{Dice}$$

where α=0.5, $L_{CE}$ is Cross-Entropy Loss [74] and $L_{Dice}$ is Dice Loss [73]. We used the ITK toolbox [75,76] for implementing the coarse and fine registration algorithms to obtain ${}^{CT_{post}}T_{CT_{pre}}$ and ${}^{F_{post}}T_{F_{pre}}$ respectively. We used normalised correlation [77] as the image similarity metric and Regular Step Gradient Descent [78] as the optimizer and assumed a rigid-body transformation with 6 degrees of freedom as our underlying transformation. The hyperparameters for the coarse registration were the following: number of iterations = 200, translation scale = 1/2000, rotation scale = 1, maximum step length = 1 and minimum step length = 0.001.

Fine alignment: The goal of the fine registration was to obtain the transformation ${}^{F_{post}}T_{F_{pre}}$ of the isolated bone fragment between pre- and postoperative position. A second registration mask was used to isolate the fragment and constrain the registration process (Figure 3(A.2)). This was achieved by first finding an approximation of the joint center *C* by computing the mean of all 4 plane centers $P_{j}$, j∈[1…4] to then ensure the plane normals $\vec{N_{j}}$ were pointing towards the approximated joint center *C*. We defined a voxel $V_{i}$ in $M_{coarse}$ as part of the fragment, if for all planes the dot product $\vec{P_{j}V_{i}}\cdot \vec{N_{j}}$ > 0, where $\vec{P_{j}V_{i}}$ is the vector pointing from the plane center $P_{j}$ to voxel $V_{i}$. The final fragment alignment is expressed by the 3 degrees of freedom (DOF) rotation and 3 DOF translation encoded in the transformation ${}^{F_{post}}T_{F_{pre}}$ obtained from the fragment alignment registration (Figure 3B, Fragment Alignment). The hyperparameters for the fine registration were the following: number of iterations = 200, translation scale = 1/500, rotation scale = 1, maximum step length = 0.7 and minimum step length = 0.0001.

#### 2.3.3. Implant Quantification

**Figure 4:**
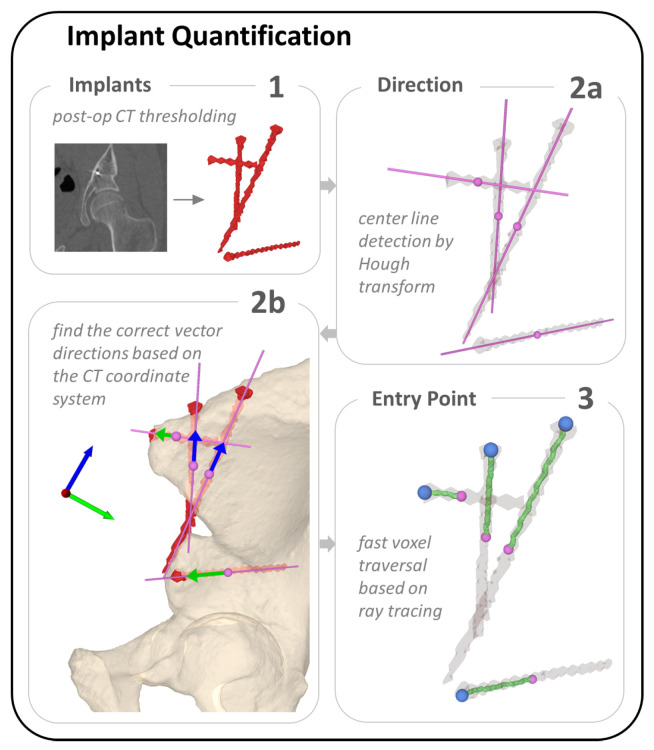
An overview of the three steps towards implant quantification. (**1**) thresholding the postoperative CT to find the screw point clouds. (**2a**) The point clouds from (**1**) are the input for the Hough transform algorithm, which finds the screw axis and center point. In (**2b**) the vector directions are verified to point towards the screw entry points using the CT coordinate system. (**3**) Entry points are determined by fast voxel traversal based on ray tracing. The center point found through Hough transform (shown in pink) is the starting point for ray tracing along the screw axis (green) until reaching the end of the screw (blue).

In PAO, multiple screws are implanted in close proximity to each other. The segmented screw mask resulting from the CT images requires isolation of each screw, which is particularly challenging. Our method of implant quantification is based on the identification of simple geometric shape features of the implants, which allow us to subsequently determine their position. In our case, the shape features are lines corresponding to the screw threads. For other implants such as osteosynthesis plates or prostheses, the shape features would be circles or spheres corresponding to the plate holes or prosthesis heads, respectively. Breaking down implant quantification to simple shape features makes Hough transform the algorithm of choice. To quantify screw location, the center line and entry point for each screw were determined in three steps (Figure 4). For center line detection, we followed the Hough transform implementation for 3D line detection based on 3D point clouds published by Dalitz et al. [69] (Figure 4(2a)). Our input were the point clouds of the screws, which we obtained by thresholding the postoperative CTs at Hounsfield unit (HU) > 2500 to find the region corresponding to metal implants (Figure 4(1)). The method was applied for each patient individually. Each point cloud included 4 to 6 screws depending on the patient case (Figure 4(2b)). In the following, we briefly summarise their method: 3D Hough transform is applied to transform each point cloud into a voting array in the parameter space [79]. A common approach to find the object in this voting array, in our case a 3D line, is to search for local maxima, also known as a “non maximum suppression” [80] which can lead to the prediction of many nearby lines. To avoid that, the Hough transform is applied iteratively, while points of detected lines are removed after each iteration. The algorithm can be adjusted for best predictions by setting the following three parameters. (1) nlines: the maximum number of lines to be detected, (2) minvotes: the minimum vote count to be detected as lines and (3) dx: the xy step width. Optimal results for our data set were found using the following parameters, which were determined experimentally: nlines=6, minvotes=50 and dx=3. With these settings, we achieved an accurate line prediction per screw (Figure 4(2a) directions and center points in pink). The algorithm returns the detected lines in a list including the following information: the number of points that have been assigned to the line, their center of mass and the line direction. In step three, screw entry points were identified using a fast voxel traversal method based on ray tracing [70] (Figure 4(3)). The center of mass per screw and the corresponding line direction, found through Hough transform, served as starting point and direction for the ray tracing algorithm within the thresholded segmentation masks. The anatomical coordinate system of the CT was used to ensure that the line direction was consistently pointing towards the screw head for all screws and patients. The ray travels through all foreground voxels along the predefined direction vector until it reaches the end of the segmentation mask, where the switch from foreground voxels to background happens, which in our case corresponds to the screw entry point.

## 3. Results

In the following sections, we report our results and compare them to the manual gold standard approach reported in Hoch et al. [23].

### 3.1. Osteotomy Location

**Figure 5:**
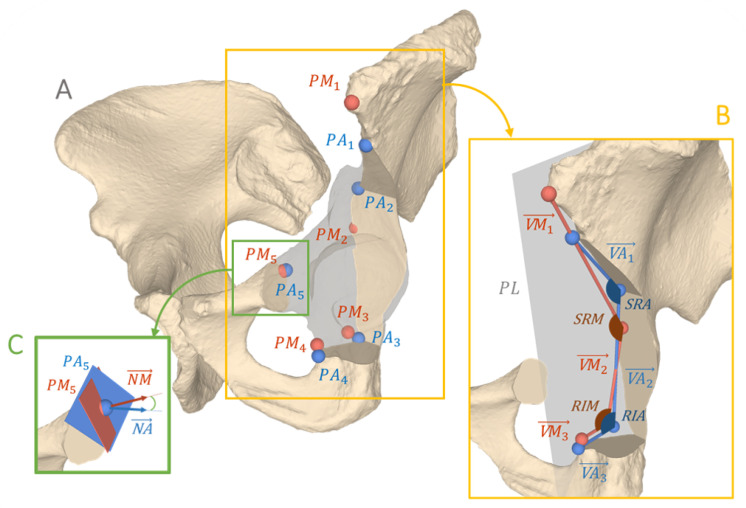
Illustration of the measures to evaluate the location of each osteotomy plane. (**A**) shows the starting point of the osteotomies $PM_{1}\ldots PM_{5}$ (manual) and $PA_{1}\ldots PA_{5}$ (automatic). (**B**) shows the projected plane PL and the connecting vectors ${\vec{VM}}_{1}\ldots {\vec{VM}}_{3}$ and ${\vec{VA}}_{1}\ldots {\vec{VA}}_{3}$ between projected points. (**C**) represents the most posterior points on plane 4, $PM_{5}$ and $PM_{4}$, as well as the normal vectors of plane 4, $\vec{NM}$ and $\vec{NA}$.

To quantify the segmentation results for osteotomy detection, we conducted a comparison against manually defined planes. Please note, that clinical gold standard for manual definition of the osteotomy planes cannot be seen as a ground truth, as it involves many manual processes that can potentially dilute the accuracy of the assessment. The following metrics were introduced for this comparison (see Figure 5A) shows the pelvic 3D model with the starting points of the osteotomies $PM_{1}\ldots PM_{5}$ (manual) and $PA_{1}\ldots PA_{5}$ (automatic), which were defined as follows:

- $PM_{1},PA_{1}$: most superior point on intersection between supraacetabular osteotomy plane and the pelvic 3D model
- $PM_{2},PA_{2}$: most medial point on intersecting line between supraacetabular and retroacetabular osteotomy planes
- $PM_{3},PA_{3}$: most medial point on intersecting line between retroacetabular and ischial osteotomy planes
- $PM_{4},PA_{4}$: most anterior point on intersection between ischial osteotomy plane and the pelvic 3D model
- $PM_{5},PA_{5}$: most posterior point on intersection between pubic osteotomy plane and the pelvic 3D model

These points were projected to a plane PL defined by the best least-squares fit of $PM_{1}\ldots PM_{4}$.

**Figure 6:**
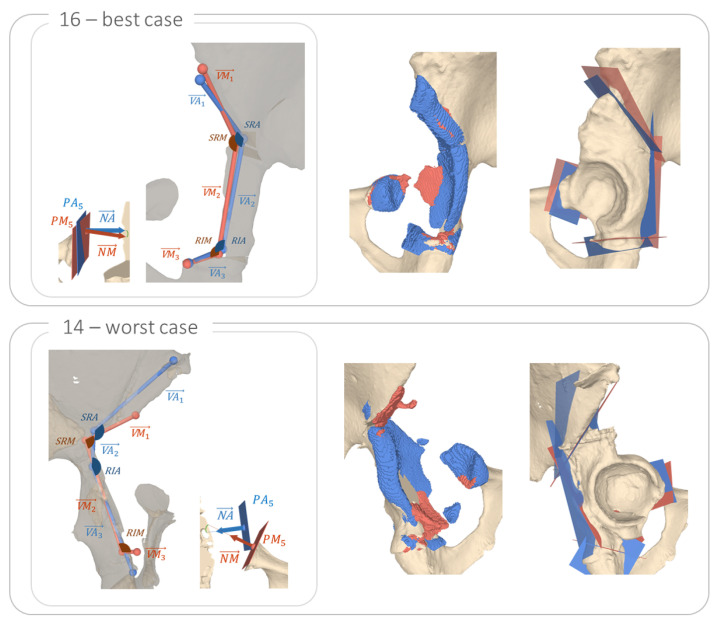
Visualisation of the best (case 16) and worst (case 14) cut detection outcome. The manual plane placement is shown in red and the automatic solution in blue.

Afterwards, the connecting vectors ${\vec{VM}}_{1}\ldots {\vec{VM}}_{3}$ and ${\vec{VA}}_{1}\ldots {\vec{VA}}_{3}$ between the projected starting points were calculated as well as the angles between them (Figure 5B). SRM (manual) and SRA (automatic) were formed by ${\vec{VM}}_{1}$, ${\vec{VM}}_{2}$ and ${\vec{VA}}_{1}$, ${\vec{VA}}_{2}$, respectively. RIM and RIA were formed by ${\vec{VM}}_{2}$, ${\vec{VM}}_{3}$ and ${\vec{VA}}_{2}$, ${\vec{VA}}_{3}$, respectively. In addition, we report the angle between the manual and automatic normal vectors $\vec{NM}$ and $\vec{NA}$ for the pubic osteotomy plane (Figure 5C). Table 1 reports the mean results over all patients, whereas individual results per patient can be found in Table A1. In Figure 6, a visualisation of the best (case 16) and worst (case 14) cut detection outcome is presented. The best and worst case were determined based on the deviation from the manual planning across all measures reported in Table A1.

### 3.2. Fragment Reorientation

**Figure 7:**
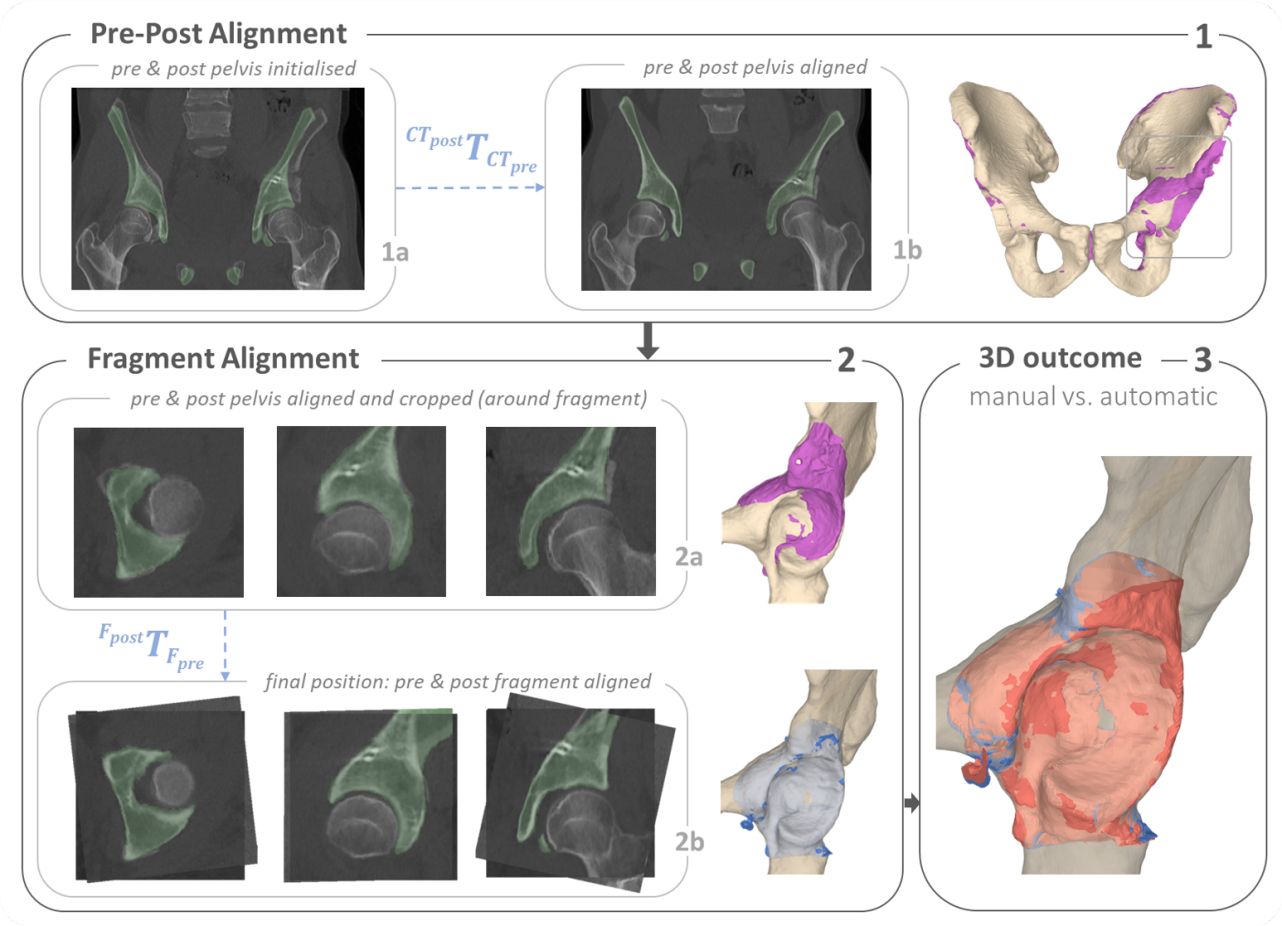
Results of quantifying anatomy repositioning for a typical case (i.e., case 19). In (**1a**–**2b**), the preoperative CT (pelvis highlighted in green) and postoperative CT images are superimposed before and after the either registration step (i.e., (**1a**): before pre-post alignment and (**1b**): after pre-post alignment). (**1**): Pre-Post Alignment. (**1a**) shows the overlay of the pre- and postoperative CTs after initialisation, which is the starting position for the pre-post alignment. (**1b**) shows the end position of the pre-post alignment, where the pre- and postoperative CTs are aligned. (**2**): For Fragment Alignment, the CT images are cropped around the fragment. (**2a**) shows the starting position for the second registration, which is found in 1. (**2b**) shows the final position, where the pre and post fragment are aligned. (**3**) shows the end position for the manual and automatic fragment alignment. The 3D postoperative bone model is shown in violet, the automatic solution is shown in blue and the manual result in red.

**Figure 8:**
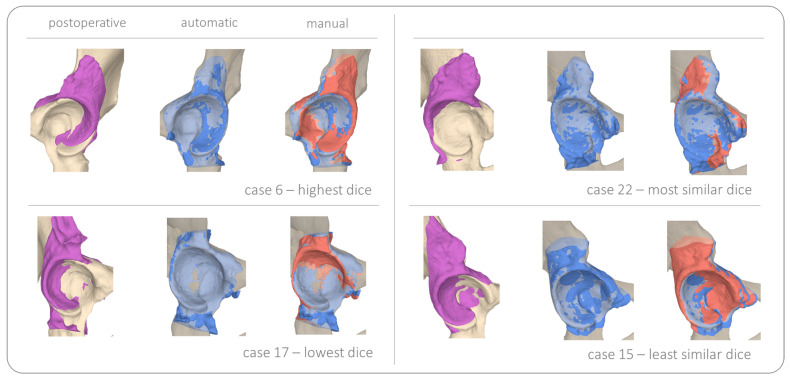
Registration results for four example cases, the postoperative bone is shown in violet, the automatically transformed fragment is shown in blue and the manually registered fragment is displayed in red. Case 6 with the highest overall dice score of 0.74, case 17 with the lowest dice score for the automatic solution of 0.47, case 22 with the most similar dice of 0.57 for both solutions and case 15 with the least similar dice scores for the automatic and manual solution, 0.65 and 0.58 respectively.

We evaluated both registration processes the pre-post alignment ${}^{CT_{post}}T_{CT_{pre}}$ and fragment alignment ${}^{F_{post}}T_{F_{pre}}$ for the manual and automatic solution by calculating the mean absolute error (MAE) between corresponding mesh points in the end position. For the first stage of registration, the coarse alignment, we found an error $err_{T1}$ of 1.01 ± 0.46 mm. In the final stage of measuring the bone fragment repositioning, we found an error $err_{T2}$ of 2.10 ± 0.97 mm. In addition, we report the dice coefficient of the postoperative CT (cropped around the acetabulum) with the preoperative CT in its final position, after applying both transformations ${}^{CT_{post}}T_{CT_{pre}}$ and ${}^{F_{post}}T_{F_{pre}}$. The mean dice coefficient across all patients for the automatic registration was $DC_{a,mean}$ 0.62 ± 0.07 and $DC_{m,mean}$ 0.60 ± 0.07 for the manual registration. In Table A2 we report the registration results for all patients, as well as the difference of dice coefficients for the automatic and manual solution $DC_{diff}=\left|DC_{a}-DC_{m}\right|$. As an ablation study, we performed 5-fold cross validation to evaluate the performance of the pelvic segmentation network. The mean dice coefficient (DC) across all folds was 0.93 ± 0.02. In Figure 7 we present the complete registration results for an example case (i.e., case 19). Figure 8 shows the 3D overlay visualisation of the registration results for four examples. Case 6 was found to be the best case ($DC_{a}$ of 0.74) and case 17 the worst case ($DC_{a}$ of 0.47). In addition case 22 and 15 are presented, which were determined to have the most and least similar dice scores for the automatic compared with the manual solution.

### 3.3. Implant Placement

**Figure 9:**
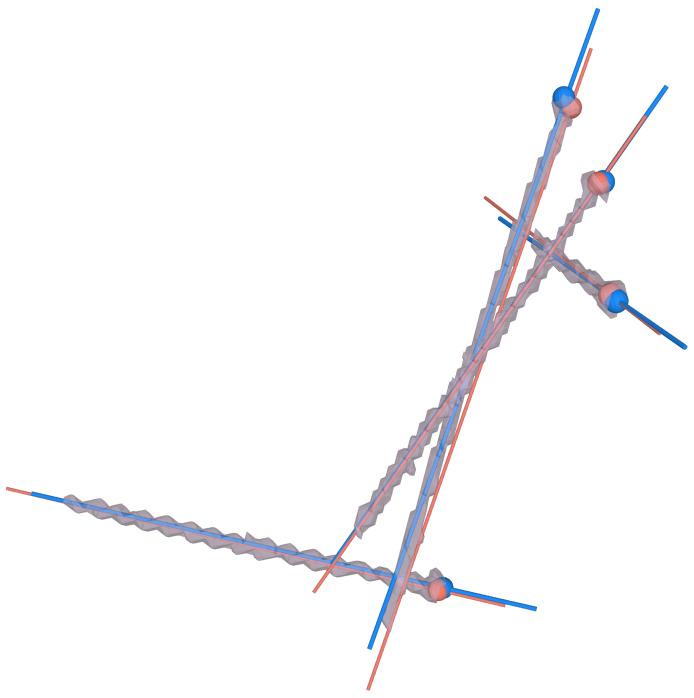
Example of a manual (red) and automatic (blue) prediction of the screw head location and center line.

We found that the mean error between manually and automatically detected screw head centers was 1.32 ± 0.49 mm. Similarly, the mean 3D angle between the screw center lines derived based on the automatic vs. the manual process was 1.10 ± 0.87°. Figure 9 shows an example of the screw head and center line prediction.

## 4. Discussion

In this study we proposed a fully automatic method for postoperative quantification of CT images after PAO interventions and compared it to manual state-of-the-art methods.

As per our knowledge, the presented method is the first to quantify osteotomy location in CT images. For cut detection, we found a mean error of projected plane starting points ($|PM_{i}-PA_{i}|$, i=1…4) for planes 1–3 of 13 ± 3.6 mm and a mean difference between 2D angle of 11.9 ± 7°. For the pubic osteotomy (plane 4), we report a 8.7 mm mean error and a 29.3° angular deviation. Kulyk et al. [15] and Tschannen et al. [22] presented methods to detect the articular marginal plane (AMP) of the proximal humerus in CT images. Tschannen et al. [22] found a 2.40 mm error in estimating the AMP center and a 6.51° mean angular error for estimating the normal vector compared to the manually annotated ground truth. Kulyk et al. [15] reported a 1.30 ± 0.65 mm mean localisation error and a 4.68 ± 2.84° angular error. Our results are inferior to those found in both, Kulyk et al. [15] and Tschannen et al. [22]. However, our results were in line with the ones found in Ackermann et al. [8], who investigated the postoperative outcome compared to the planned osteotomies for PAO. During a typical PAO intervention, only plane 1 is cut with a surgical saw whereas plane 2 to 4 are performed with a chisel. Furthermore, the crossing between plane 2 and plane 3 (angle RIM and RIA) is achieved by a controlled fracture, which makes a true plane fit, specifically the distinguishing between plane 2 and 3, more complex since it often presents as a curvature rather than two individual planes intersecting. Generally larger errors in cut prediction were found for planes with small surface areas (i.e., the pubic osteotomy, plane 3 and the iscial osteotomy, plane 4). One example is case 14, where the cut surfaces of plane 1 and 3 are forming a gap and only small areas of the bone fragments touch and form a callus, as can be seen in Figure 6. Moreover, the training data for the segmentation network was not annotated based on the manual plane detection but rather based on callus formation.

The evaluation of our fragment repositioning showed a 1.01 mm mean point-to-point distance error for the pre- to postoperative CT registration and 2.10 mm in the final position of the acetabular fragment. In 2011, Murphy et al. [81] compared 20 individual registration algorithms on a set of 30 intra-patient thoracic CT image pairs and found a mean error for landmark alignment of 0.83 mm for the best 6 algorithms. Their mean voxel size was 0.7 mm and a registration mask was used. Although the set of images used in Murphy et al. [81] had larger variety of information deviation between image pairs (due to respiratiory changes), we argue that our results are in a similar range and therefore acceptable. Moreover, $err_{T12}$[mm] reports the combined error of both registration processes. It compares the starting position of the preoperative bone model to the end position of the acetabular fragment. Interestingly, although the largest deviation between meshes in the end position $err_{T12}$[mm] lwas found for case 6, the best dice score $DC_{a}$ was achieved for that case, which suggest best overlap with the preoperative bone mesh. This can also be confirmed visually, shown in Figure 8. As reported in Table A2, the mean dice score for the automatic approach $DC_{a}$ was found to be slightly superior to the manual method $DC_{m}$. Furthermore, no correlation was found between the performance of the segmentation network for the registration mask and the registration result.

For quantification of the screw positions, we found a 1.32 mm MAE for the screw head prediction and a 1.10° screw axis error. In 2013, Uozumi et al. [45] published an automatic 3D approach for screw placement evaluation where they first isolated the screw point clouds through thresholding the images and reported the center of mass. Then they applied PCA to find the screw axis. They found a 0.14 mm mean distance error and a 0.02° average angular error, which is superior to our results. Uozumi et al. [45] achieved higher accuracy with their method because their screw point clouds were isolated for each screw, therefore identifying the center of mass and direction was a straight forward process. In our case however, the screws are very close to each other, which results in point clouds combining multiple screws which is a more complex task, hence the lower accuracy. Moreover, our results are superior to the pedicle screw placement accuracy reported in Jiang et al. [82] and Gubian et al. [44], which were both considered clinically acceptable. Jiang et al. [82] evaluated robot-assisted pedicle screw placement and found a mean screw tip accuracy of 3.6 ± 2.3 mm and an angular deviation of 3.6 ± 2.8°. Gubian et al. [44] evaluated CT navigated pedicle screw placement by comparing the preoperative trajectory with the corresponding postoperative screw position and found a mean displacement of 5.2 ± 2.4 mm for the screw head points and a mean axis deviation of 6.3 ± 3.6°.

Our work has several limitations. A drawback of our method is the radiation exposure during CT acquisition. Moreover, the manual data labelling required for training is very time consuming. However, with the introduction of low-dose CTs less harmful imaging will replace conventional methods in the future [83]. Furthermore, recent AI based reconstruction algorithms can be leveraged, which are capable of generating accurate 3D models from X-ray or fluoroscopy data [13,84]. Confounders which are difficult to measure can be introduced at different steps of our pipeline. First of all, we compare our results to a manual approach, which may not correspond the ground truth measurements. Other potential sources might be the manual segmentation and the resampling of the images at several points throughout the pipeline. In addition, no inter-observer bias for the manual approach was investigated. Nevertheless, the large deviation of the automatic cut detection and the manual gold standard show the need for further validation studies that will be conducted in future work. An automated method would also not replace the radiology expert, but would instead represent a computer-assisted radiology approach that provides more information while saving time. The postoperative imaging for our data set was taken approximately 15 weeks post intervention, when bone healing has already occurred. We plan to investigate whether a higher standardised postoperative follow up, which takes place sooner after surgery, will result in a higher accuracy of our method. Future work will also include the generalisation of the proposed method to other orthopaedic interventions as well as being expanded to fracture detection.

## 5. Conclusions

Our method offers several advantages: Firstly, no manual input is required, which reduces the evaluation process by multiple hours per patient when compared to manual 3D analysis. Moreover, our method ensures objectivity in the assessment, providing reliable and consistent results and therefore contributes to enhancing the overall quality of treatment.

## Figures and Tables

**Table 1:** Mean difference between the manual and automatic cut detection across all patients. The measurements were calculated according to Section 2.3.1.

Measure	Manual	Automatic	Mean	σ	Min	Max
3D distance [mm]	$PM_{1}$	$PA_{1}$	17.0	13.0	2.9	54.9
	$PM_{2}$	$PA_{2}$	12.8	7.4	1.8	27.8
	$PM_{3}$	$PA_{3}$	15.0	12.1	4.3	71.4
	$PM_{4}$	$PA_{4}$	7.3	6.3	1.0	25.5
	$PM_{5}$	$PA_{5}$	8.7	6.7	1.3	23.5
2D angle [°]	${\vec{VM}}_{1}$	${\vec{VA}}_{1}$	7.0	5.4	0.3	22.2
	${\vec{VM}}_{2}$	${\vec{VA}}_{2}$	6.9	5.4	1.6	23.4
	${\vec{VM}}_{3}$	${\vec{VA}}_{3}$	21.9	17.7	0.6	73.3
Abs. 2D angle deviation [°]	SRM	SRA	9.9	8.5	0.0	36.5
	RIM	RIA	20.0	15.2	0.3	49.9
3D angle [°]	$\vec{NM}$	$\vec{NA}$	29.2	11.2	9.6	54.4

## Data Availability

The data presented in this study is available upon request.

## Appendix A

**Table A1:** Results for the comparison of manual and automatic cut detection for all patients.

Patient	3D Distance [mm]					2D Angle [°]			abs. 2D Angle		3D Angle [°]
									**Deviation [°]**		
Manual	${\mathrm{PM}}_{1}$	${\mathrm{PM}}_{2}$	${\mathrm{PM}}_{3}$	${\mathrm{PM}}_{4}$	${\mathrm{PM}}_{5}$	${\vec{\mathrm{VM}}}_{1}$	${\vec{\mathrm{VM}}}_{2}$	${\vec{\mathrm{VM}}}_{3}$	SRM	RIM	$\vec{\mathrm{NM}}$
Automatic	${\mathrm{PA}}_{1}$	${\mathrm{PA}}_{2}$	${\mathrm{PA}}_{3}$	${\mathrm{PA}}_{4}$	${\mathrm{PA}}_{5}$	${\vec{\mathrm{VA}}}_{1}$	${\vec{\mathrm{VA}}}_{2}$	${\vec{\mathrm{VA}}}_{3}$	SRA	RIA	$\vec{\mathrm{NA}}$
Mean	17.0	12.8	15.0	7.3	8.7	7.0	6.9	21.9	9.9	20.0	29.2
σ	13.0	7.4	12.1	6.3	6.7	5.4	5.4	17.7	8.5	15.2	11.2
0	32.4	25.8	9.9	6.4	2.0	11.6	10.5	0.6	1.1	11.1	29.5
1	3.3	5.7	10.9	8.3	5.1	2.2	8.9	1.0	11.1	9.9	21.6
2	11.2	7.3	9.4	2.1	6.2	8.8	3.5	3.2	5.2	0.3	31.2
3	20.6	10.2	6.7	1.2	8.2	12.1	2.3	8.5	9.8	6.2	20.0
4	12.4	8.2	14.2	8.7	17.7	0.4	2.0	14.0	1.6	16.0	48.5
5	6.6	10.5	15.0	5.9	21.2	2.9	2.9	14.7	0.0	11.8	49.1
6	26.7	7.8	17.3	7.4	1.3	2.4	2.8	39.7	0.4	36.9	19.2
7	14.5	27.8	13.1	5.6	3.4	3.0	18.9	19.1	15.9	0.3	25.3
8	3.3	18.8	7.0	2.6	16.9	7.8	1.9	17.6	5.9	15.7	54.4
9	10.9	16.5	11.8	25.5	21.1	1.2	7.0	43.1	8.2	36.0	49.3
10	14.6	2.6	11.4	5.3	3.6	5.1	3.9	26.3	9.0	22.4	28.5
11	16.8	26.1	16.5	6.7	10.1	7.6	17.1	15.9	9.5	1.2	36.9
12	28.2	3.2	20.0	4.2	4.3	7.6	8.6	39.5	1.1	30.9	18.4
13	14.0	10.8	6.0	1.2	15.4	9.4	5.1	12.0	14.5	17.1	28.3
14: worst	54.9	10.9	71.4	17.3	23.5	13.1	23.4	73.3	36.5	49.9	32.9
15	2.9	1.8	15.7	6.5	14.5	1.6	5.7	45.8	7.3	40.1	38.2
16: best	6.1	5.9	4.3	2.4	1.5	10.3	1.6	5.1	11.9	6.8	9.6
17	4.8	12.2	12.1	10.6	4.0	10.5	7.1	6.7	3.4	0.4	21.0
18	14.9	11.1	13.1	2.9	2.3	0.3	7.9	8.9	8.2	16.8	27.9
19	5.8	10.8	13.3	24.0	11.4	1.7	1.7	20.4	3.4	18.7	35.5
20	29.6	16.1	14.8	1.0	5.9	1.8	9.4	11.5	11.2	20.9	17.6
21	5.5	6.8	20.6	6.5	6.8	10.9	3.7	43.8	7.3	47.5	20.6
22	30.9	11.9	7.8	5.2	3.1	11.2	4.4	6.6	15.6	11.0	22.0
23	42.9	18.3	10.3	13.1	8.6	22.2	8.5	14.3	30.7	5.8	33.2
24	23.4	22.2	16.9	9.0	5.7	0.8	8.2	20.8	7.4	29.0	21.4
25	15.6	12.8	19.4	3.7	7.3	13.0	3.6	39.8	16.6	43.4	22.1
26	6.0	23.3	16.9	4.2	2.4	10.1	4.7	38.2	14.8	33.6	25.8

**Table A2:** Registration results for all patients.

Case	${\mathrm{err}}_{T1}$ [mm]	${\mathrm{err}}_{T12}$ [mm]	${\mathrm{DC}}_{m}$	${\mathrm{DC}}_{a}$	${\mathrm{DC}}_{\mathrm{diff}}$
$mean_{all}$	1.01	2.10	0.60	0.62	0.02
σ	0.46	0.97	0.07	0.07	0.02
0	0.38	1.4	0.48	0.48	0.003
1	0.42	2.48	0.67	0.7	0.036
2	1.47	1.61	0.71	0.71	0.002
3	1.43	2.07	0.71	0.74	0.027
4	0.79	1.86	0.62	0.66	0.039
5	0.51	2.46	0.58	0.59	0.016
6: $DC_{a,max}$	0.71	5.84	0.68	0.74	0.059
7	1.83	2.25	0.61	0.64	0.028
8	1.25	1.76	0.62	0.67	0.048
9	0.86	1.64	0.62	0.64	0.017
10	1.57	1.92	0.63	0.64	0.003
11	0.66	1.24	0.49	0.51	0.011
12	0.41	0.55	0.62	0.64	0.025
13	1.2	2.27	0.56	0.62	0.052
14	0.54	1.72	0.57	0.6	0.034
15: $DC_{diff,max}$	1.72	2.73	0.58	0.65	0.068
16	1.69	3.27	0.71	0.7	0.004
17: $DC_{a,min}$	0.27	0.33	0.44	0.47	0.023
18	1.31	2.45	0.69	0.69	0.004
19: example	0.88	1.6	0.53	0.55	0.017
20	0.74	2.25	0.53	0.54	0.008
21	0.96	2	0.64	0.65	0.015
22: $DC_{diff,min}$	1.19	2.04	0.57	0.57	0.001
23	1.61	3	0.53	0.54	0.007
24	1.3	2.44	0.6	0.61	0.003
25	1.2	1.41	0.68	0.69	0.01
26	0.48	2.02	0.6	0.6	0.002
